# An Unusual Case of Esophageal Perforation Following C5-C7 Anterior Cervical Corpectomy and Fusion Managed Surgically With Esophageal Repair and Long-Segment Cervical Fusion

**DOI:** 10.7759/cureus.73256

**Published:** 2024-11-07

**Authors:** Tushar Pisal, Sagar Gurnani, Ajinkya K Chaudhari

**Affiliations:** 1 Department of Orthopaedics, Dr. D. Y. Patil Medical College, Hospital and Research Centre, Dr. D. Y. Patil Vidyapeeth (Deemed to Be University), Pune, IND; 2 Department of Orthopaedics, Sainath Hospital, Pune, IND

**Keywords:** anterior cervical spine surgery, cervical spine surgery complication, oesophageal perforation, postoperative dysphagia, spine surgery complication

## Abstract

Anterior cervical corpectomy and fusion (ACCF) is frequently the surgical management for myelopathy, radiculopathy, and cervical spine trauma. Although esophageal perforation is an uncommon complication, it remains a serious concern. This report details the case of a 50-year-old female who underwent a C6 corpectomy with C5-C7 ACCF due to degenerative pathology and subsequently developed an esophageal perforation that required revision surgery and surgical repair of the perforated esophagus. A comprehensive review for surgically managing esophageal perforation following cervical spine surgery is essential for intensivists to improve postoperative airway management strategies.

## Introduction

Anterior cervical corpectomy and fusion (ACCF) is frequently used to surgically manage myelopathy, radiculopathy, and cervical spine trauma [1,2]. Although esophageal perforation is an uncommon complication occurring in 0.02% to 1.52% of cases, it remains a serious concern [3]. These complications, though rare, are more common in cases of cervical trauma. We describe a case of a 50-year-old female who underwent surgical management by C6 corpectomy with C5-C7 anterior fusion following degenerative pathology and subsequently developed esophageal perforation requiring surgical intervention.

## Case presentation

**Figure 1:**
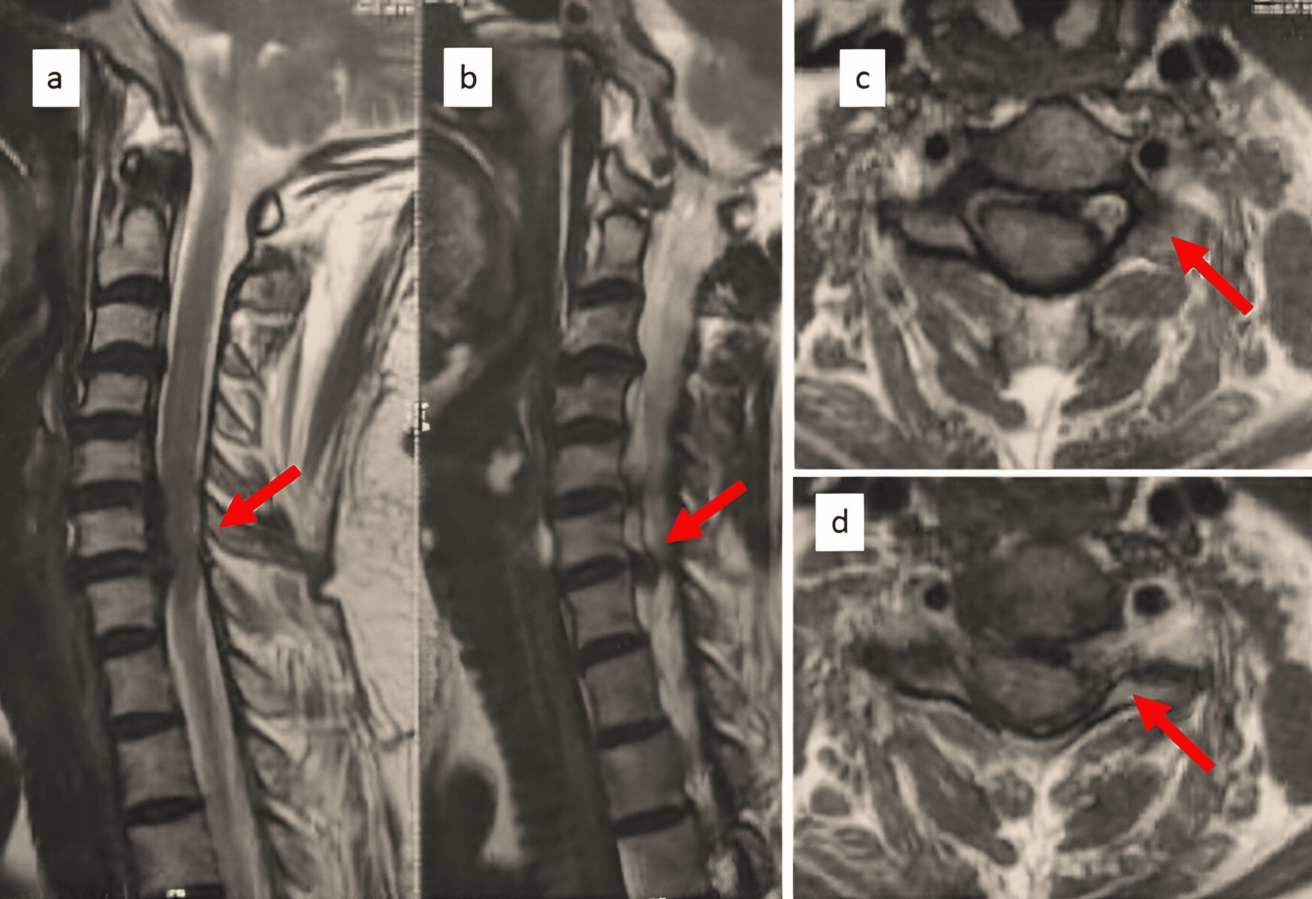
Preoperative MRI showing C5-C6 and C6-C7 prolapsed intervertebral disc. Preoperative MRI images of the cervical spine: (a-b) sagittal cut with the red arrow highlighting disc pathology; (c-d) axial MRI cut with the red arrow highlighting disc pathology.

**Figure 2:**
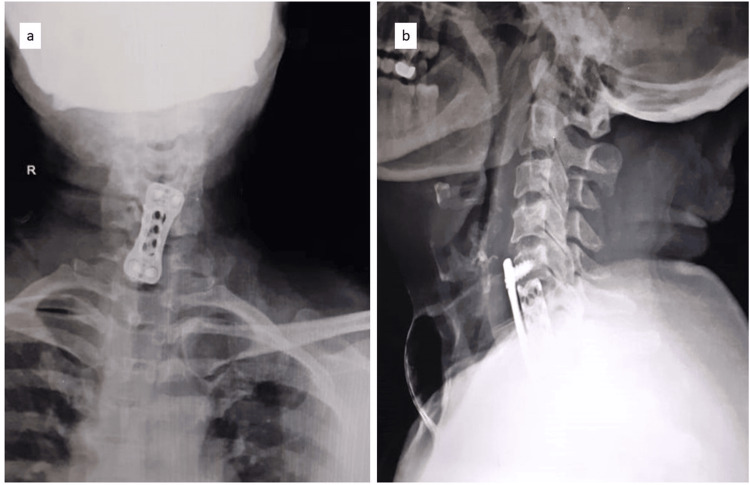
Postoperative radiographs after ACCF of the cervical spine. Postoperative radiographs showing C6 corpectomy with C5-C7 fusion, MESH cage, and anterior cervical plate; (a) anteroposterior radiograph, (b) lateral radiograph ACCF: anterior cervical corpectomy and fusion

**Figure 3:**
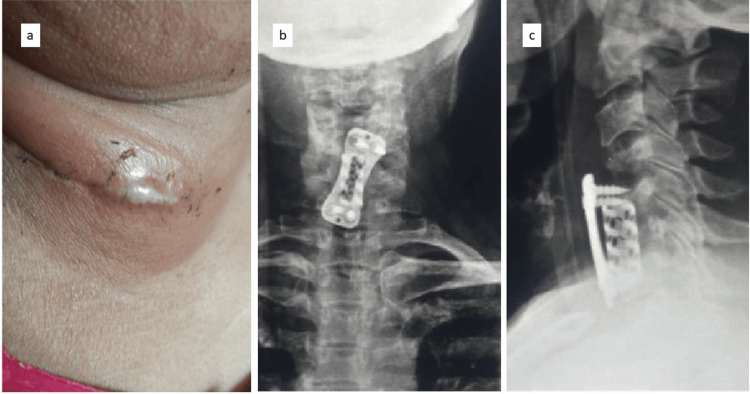
Clinical and radiographic picture of the patient at three weeks postoperatively. (a) Discharging sinus at the surgical site; (b) anteroposterior radiograph at three weeks postoperatively; (c) lateral radiograph at three weeks postoperatively

A 50-year-old female presented to our hospital with neck pain radiating to the bilateral upper limbs, decreased finger grip, and signs of myelopathy. MRI showed spinal cord compression at the C5-C6 and C6-C7 levels (Figure 1). The patient underwent anterior cervical decompression and fusion, along with a C6 corpectomy with a MESH cage and local bone graft to aid fusion at the C5-C7 level (Figure 2). Postoperatively, the patient responded well to the surgery; hand grip and gait improved along with neck pain and radiculopathy. Suture removal was done postoperatively on day 12. The patient began postoperative rehabilitation and was immobilized with a soft cervical collar. Approximately three weeks after surgery, she presented with a discharging sinus from the surgical site. She was managed conservatively with dressing and antibiotics (Figure 3).

**Figure 4:**
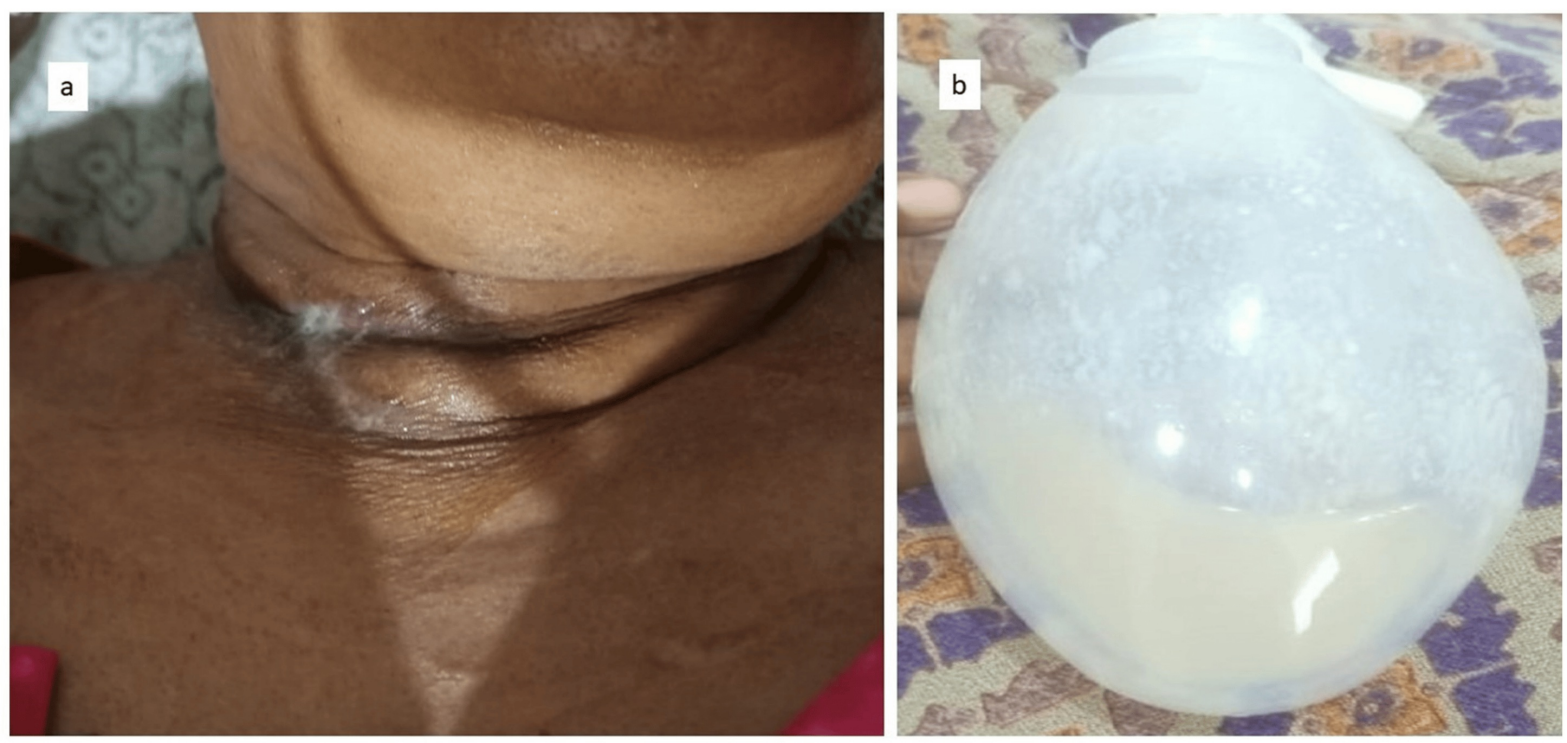
Sinus burst open at four weeks, thorough debridement was done, and a drain was inserted. The sinus burst open at four weeks and the patient underwent debridement and a drain was inserted to let all the collected debris flow out; (a) clinical photo of the sinus at four weeks; (b) drain and collection in the drain.

The sinus reappeared after a week (Figure 4) and the patient was planned for a thorough local debridement in the operating theatre. Intraoperative pus samples were taken, the wound was sutured and a drain was placed to collect secretions (Figure 4). The collected sample tested positive for *Candida tropicalis* (Table 1). The patient was started on intravenous caspofungin, which was not well tolerated, and was then shifted to intravenous amphotericin B for a duration of two weeks. The patient was then shifted to oral fluconazole for six weeks. On day 45 post-surgery, swallowed liquid began to secrete from the wound. A barium study suggested an esophageal fistula at the infection site. The patient was immediately placed on Ryle's tube feeding, which continued for five weeks. The Ryle’s tube was removed after five weeks and the sinus was in the healing stage.

**Table 1:** Fungal culture and sensitivity report The culture sensitivity report of the intraoperative sample showing *Candida tropicalis* infection and the antibiogram for the organism. MIC: minimum inhibitory concentration

Antifungal Agent	MIC Value (microgram/millilitre)	Interpretation
Amphotericin B	≤0.25	Sensitive
Caspofungin	≤0.12	Sensitive
Fluconazole	1	Sensitive
Flucytosine	≤1	Sensitive
Micafungin	≤0.06	Sensitive
Voriconazole	≤0.12	Sensitive

**Figure 5:**
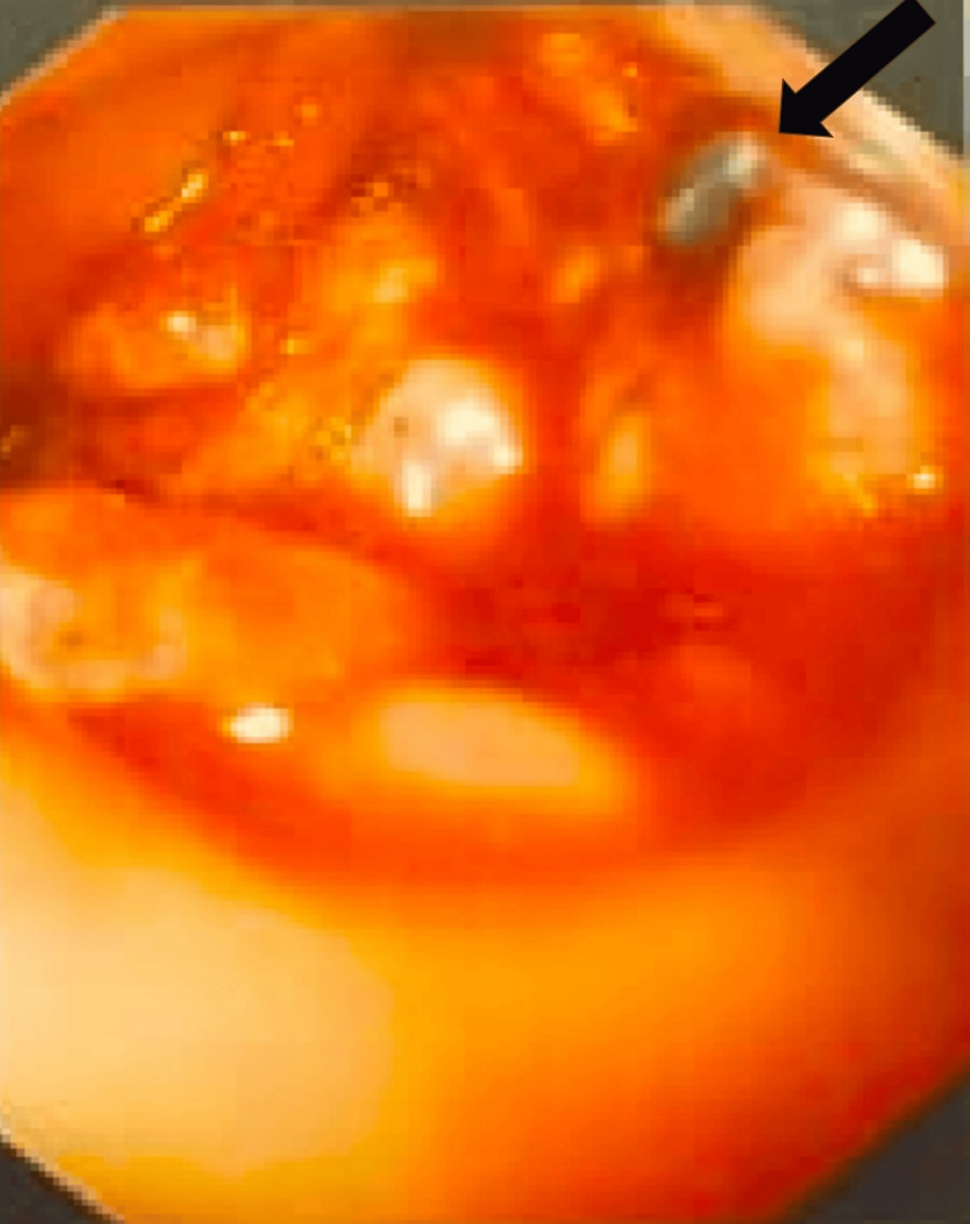
Esophageal fistula seen on endoscopy and implant perforating the esophagus. The patient underwent a diagnostic esophageal perforation; the black arrow showing esophageal perforation and implant seen on endoscopy.

**Figure 6:**
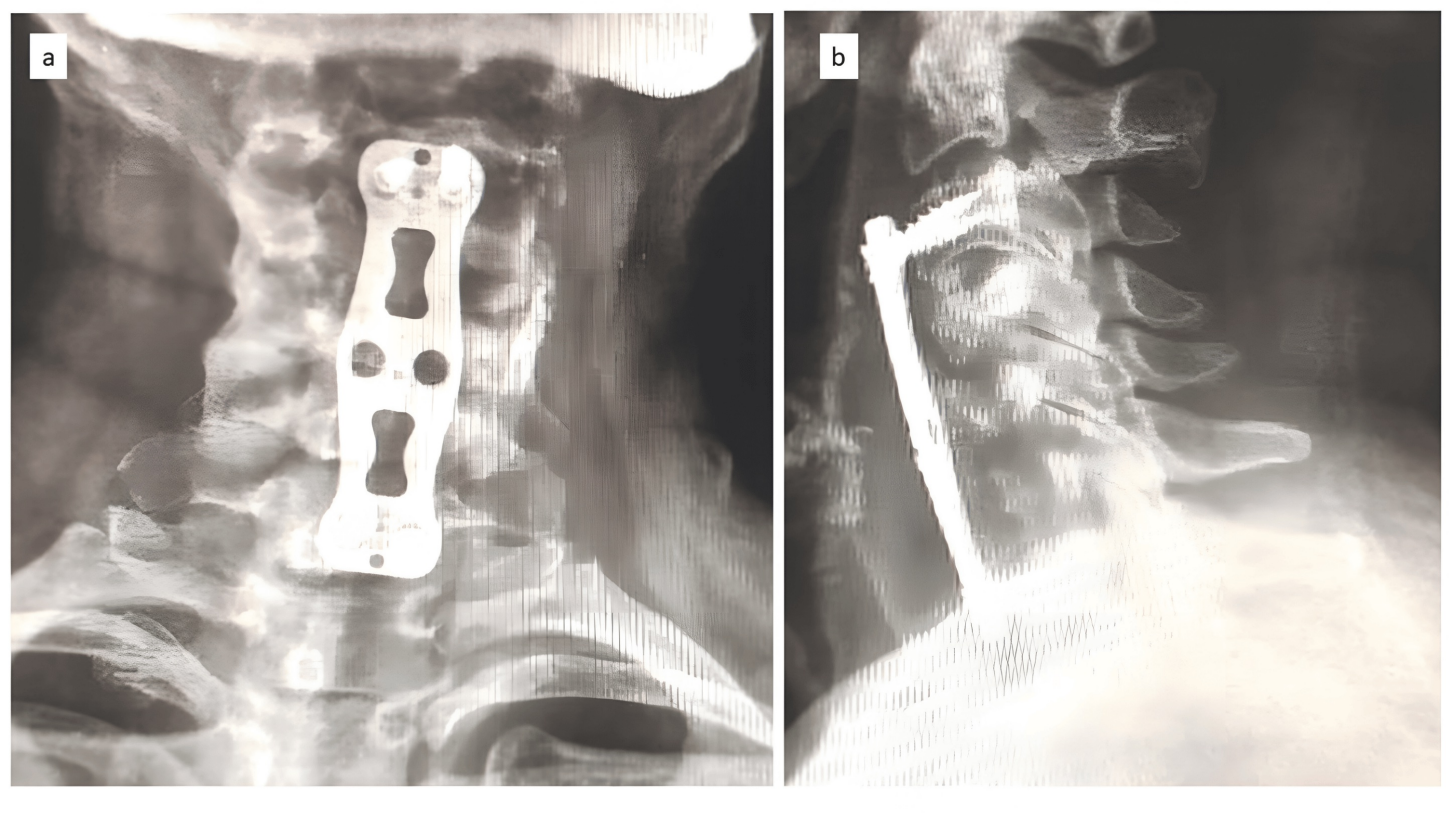
Postoperative radiographs after revision surgery showing extension of the fusion from C3 to C7. The patient underwent repair of the esophageal perforation and long-segment fusion from C3 to C7; (a) anterolateral postoperative radiograph and (b) lateral postoperative radiograph

**Figure 7:**
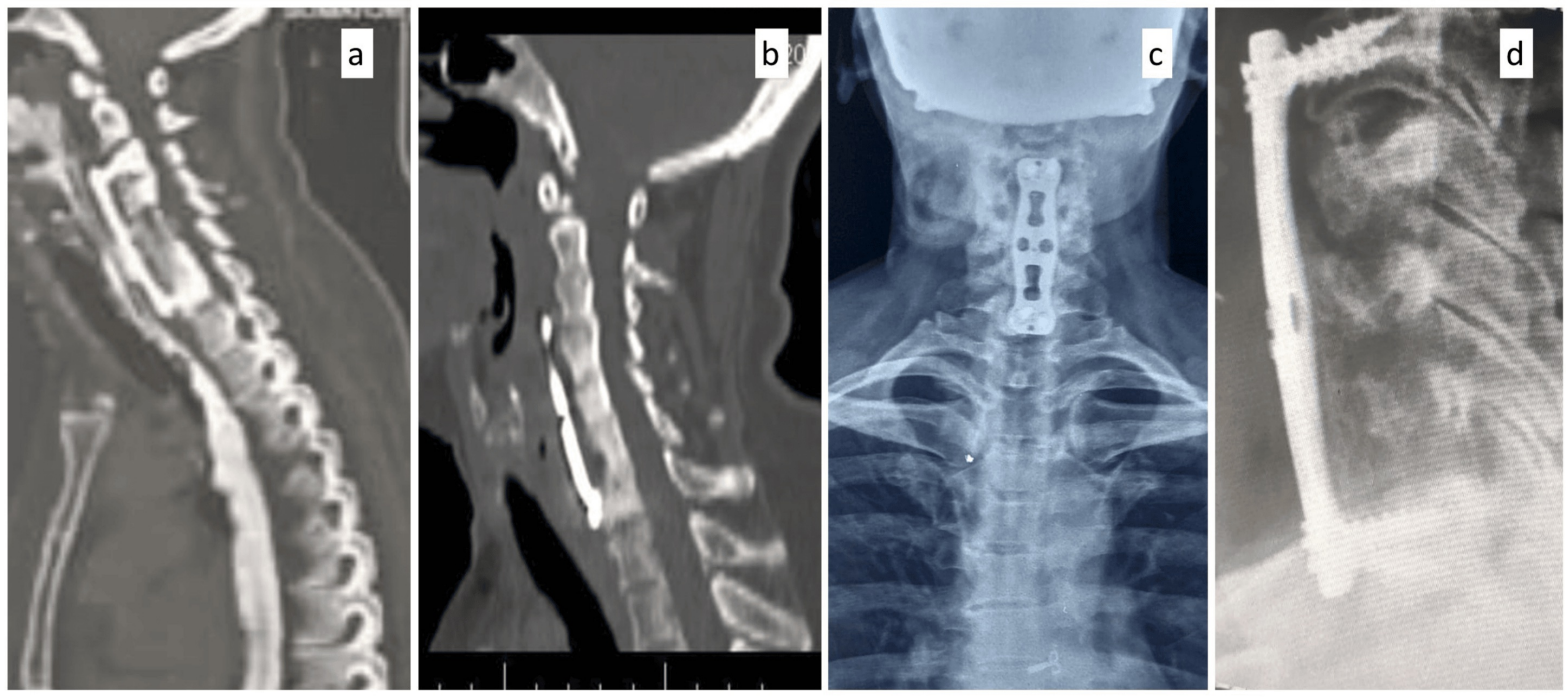
Postoperative computer tomography scans and radiographs after revision surgery showing extension of the fusion from C3 to C7 six weeks postoperatively. The patient was re-evaluated six weeks after the second surgery with a computer tomography scan (CT scan) and radiographs; (a-b) sagittal CT image, (c) anteroposterior radiograph, and (d) lateral radiograph at six weeks postoperatively.

The patient was started on oral feeding, which again led to leakage from the wound after one week. Hence, as a better modality of investigation, an upper gastrointestinal endoscopy was planned for the patient. An upper gastrointestinal endoscopy suggested the implant was herniating through the esophageal tear (Figure 5). The patient underwent implant removal; the esophageal tear was identified and repaired with braided and coated polyglactin 910 (Ethicon, Inc., Somerville, USA) under vision and necrotic bone pieces were removed. The fusion segment was extended from C3 to C7 using an iliac crest bone graft along with an anterior plate (Figure 6). At four weeks of follow-up, the wound was healed, the retained tube (RT) was removed, and the patient had no dysphagia or other complaints, suggesting the esophageal injury had healed. This was confirmed by a modified barium swallow, which showed no abnormalities. The patient was on the six-week follow-up after the second surgery (Figure 7). The patient was immobilized for 12 months with a soft cervical collar.

## Discussion

For a long time, neurosurgeons and orthopedic surgeons have used ACCF to surgically manage neurological symptoms and pain that do not respond to conservative treatments. The anterior approach has excellent clinical outcomes with low mortality and complication rates for surgically managing cervical spine pathologies effectively and safely [4-7]. The complication rate of anterior cervical procedures is 13.2%, though minor and requiring little to no additional intervention [8]. The major complications in ACCF are injury to recurrent laryngeal nerve, soft tissue swelling, local hematoma, cerebrospinal fluid leakage, carotid artery injury, root and spinal cord injury, tracheoesophageal injury, and superficial wound infection [4,5,9,10]. Esophageal perforation has an incidence ranging from 0.25% to 1.49% from all causes [11], while the global incidence specifically after ACCF is reported to be between 0% and 3.4% [4]. The infrequency and varied incidence of esophageal perforation make it a challenging diagnosis that needs clinical suspicion to investigate and discover the underlying complication. The most common cause of esophageal perforation following spine surgery is hardware failure comprising plate migration, screw migration, and loosening of components. This is followed by other causes like chronic hardware erosion, intraoperative injury, graft extrusion, and penetration [12].

Given the anatomical positioning of the cervical spine posterior to the esophagus, retracting and mobilization of the esophagus are necessary to gain access to the spine during ACCF [13]. The esophagus has multiple layers, which need to be compromised before a complete perforation making it a rare complication [13]. The most common causes of esophageal perforation during ACCF are intraoperative manipulation and retraction, with hardware failure being another significant cause [11]. Postoperative esophageal perforation intensive treatment is required and typically involves additional surgery, which can lead to serious complications [5,10]. Delayed esophageal injuries can result from chronic irritation, pressure from the implants, and continuous friction between the esophagus’s posterior wall and the plating system. In cases of acute esophageal rupture, infection can significantly contribute to post-anterior cervical spine surgery. The authors hypothesize that since the fixation in the index surgery was optimal, the chance of hardware failure is less based on intraoperative and postoperative findings. In this case, the early postoperative presentation of symptoms suggests that a post-fungal infection is the most likely cause of the extrusion of the screw and esophageal rupture.

The size of the esophageal perforation decides the treatment [7]. For defects less than 1 cm in asymptomatic patients, antibiotics or other non-surgical treatments are recommended [6]. The gold standard for perforation of more than 1 cm or in cases with local infection is surgical management. The surgical intervention typically involves draining the abscesses, removing implants, and repairing the perforation [6]. Given that delays in diagnosis and treatment can significantly increase morbidity and mortality, it is crucial to complete the workup promptly when a perforation is suspected, particularly in patients with persistent dysphagia [14].

## Conclusions

Early-onset esophageal perforation post-anterior cervical surgery is extremely rare and has a variable presentation that makes the diagnosis challenging for the orthopedic surgeon. The operating surgeon should always keep it in the back of the mind when the patient presents with dysphagia or local infection postoperatively after ACCF. It is necessary to send cultures for fungal sensitivity along with bacterial culture sensitivity. Although less common, proper drug sensitivity testing is critical for effective treatment.

## References

[1] Fengbin Y, Xinwei W, Haisong Y, Yu C, Xiaowei L, Deyu C (2013). Dysphagia after anterior cervical discectomy and fusion: a prospective study comparing two anterior surgical approaches. Eur Spine J.

[2] Gaudinez RF, English GM, Gebhard JS, Brugman JL, Donaldson DH, Brown CW (2000). Esophageal perforations after anterior cervical surgery. J Spinal Disord.

[3] Hershman SH, Kunkle WA, Kelly MP (2017). Esophageal perforation following anterior cervical spine surgery: case report and review of the literature. Global Spine J.

[4] Shriver MF, Lewis DJ, Kshettry VR, Rosenbaum BP, Benzel EC, Mroz TE (2017). Dysphagia rates after anterior cervical discectomy and fusion: a systematic review and meta-analysis. Global Spine J.

[5] Tasiou A, Giannis T, Brotis AG (2017). Anterior cervical spine surgery-associated complications in a retrospective case-control study. J Spine Surg.

[6] Eroglu A, Turkyilmaz A, Aydin Y, Yekeler E, Karaoglanoglu N (2009). Current management of esophageal perforation: 20 years experience. Dis Esophagus.

[7] Fountas KN, Kapsalaki EZ, Machinis T, Robinson JS (2006). Extrusion of a screw into the gastrointestinal tract after anterior cervical spine plating. J Spinal Disord Tech.

[8] Nathani A, Weber AE, Wahlquist TC, Graziano GP, Park P, Patel RD (2015). Delayed presentation of pharyngeal erosion after anterior cervical discectomy and fusion. Case Rep Orthop.

[9] Nourbakhsh A, Garges KJ (2007). Esophageal perforation with a locking screw: a case report and review of the literature. Spine (Phila Pa 1976).

[10] Yang SY, Lee SB, Cho KS (2015). Delayed esophagus perforation after anterior cervical spine surgery. Korean J Neurotrauma.

[11] Newhouse KE, Lindsey RW, Clark CR, Lieponis J, Murphy MJ (1989). Esophageal perforation following anterior cervical spine surgery. Spine (Phila Pa 1976).

[12] Halani SH, Baum GR, Riley JP, Pradilla G, Refai D, Rodts GE Jr, Ahmad FU (2016). Esophageal perforation after anterior cervical spine surgery: a systematic review of the literature. J Neurosurg Spine.

[13] Yue WM, Brodner W, Highland TR (2005). Persistent swallowing and voice problems after anterior cervical discectomy and fusion with allograft and plating: a 5- to 11-year follow-up study. Eur Spine J.

[14] Zhong ZM, Jiang JM, Qu DB (2013). Esophageal perforation related to anterior cervical spinal surgery. J Clin Neurosci.

